# CD40L-Tri, a novel formulation of recombinant human CD40L that effectively activates B cells

**DOI:** 10.1007/s00262-012-1331-4

**Published:** 2012-08-25

**Authors:** Masayasu Naito, Ursula Hainz, Ute E. Burkhardt, Buyin Fu, Deborah Ahove, Kristen E. Stevenson, Mohini Rajasagi, Baogong Zhu, Anselmo Alonso, Elizabeth Witten, Ken-ichi Matsuoka, Donna Neuberg, Jonathan S. Duke-Cohan, Catherine J. Wu, Gordon J. Freeman

**Affiliations:** 1 Cancer Vaccine Center, Dana-Farber Cancer Institute, 44 Binney Street, Boston, MA USA; 2 Department of Medical Oncology, Dana-Farber Cancer Institute, Boston, MA USA; 3 Department of Biostatistics and Computational Biology, Dana-Farber Cancer Institute, Boston, MA USA; 4 Department of Medicine, Harvard Medical School, Boston, MA USA

**Keywords:** CD40, CD40L, B lymphocytes, Immunotherapy, Antigen-presenting cell

## Abstract

CD40L has a well-established role in enhancing the immunostimulatory capacity of normal and malignant B cells, but a formulation suitable for clinical use has not been widely available. Like other TNF family members, in vivo and in vitro activity of CD40L requires a homotrimeric configuration, and growing evidence suggests that bioactivity depends on higher-order clustering of CD40. We generated a novel formulation of human recombinant CD40L (CD40L-Tri) in which the CD40L extracellular domain and a trimerization motif are connected by a long flexible peptide linker. We demonstrate that CD40L-Tri significantly expands normal CD19+ B cells by over 20- to 30-fold over 14 days and induces B cells to become highly immunostimulatory antigen-presenting cells (APCs). Consistent with these results, CD40L-Tri-activated B cells could effectively stimulate antigen-specific T responses (against the influenza M1 peptide) from normal volunteers. In addition, CD40L-Tri could induce malignant B cells to become effective APCs, such that tumor-directed immune responses could be probed. Together, our studies demonstrate the potent immune-stimulatory effects of CD40L-Tri on B cells that enable their expansion of antigen-specific human T cells. The potent bioactivity of CD40L-Tri is related to its ability to self-multimerize, which may be facilitated by its long peptide linker.

**Electronic supplementary material:**

The online version of this article (doi:10.1007/s00262-012-1331-4) contains supplementary material, which is available to authorized users.

## Introduction

The CD40:CD40Ligand (CD40L) pathway provides essential signals for T cell help for B cell antibody production and dendritic cell priming of CD8+ T cell responses. CD40 is a member of the tumor necrosis factor receptor (TNFR) superfamily and is a type I transmembrane protein that is highly expressed on diverse cell types including many antigen-presenting cells (APC), such as B cells, dendritic cells, macrophages, and endothelial cells [1, 2]. The physiological ligand of CD40 is CD40L (CD154). Expressed on activated T cells, this type II transmembrane protein is a member of the TNF family of ligands and naturally forms a trimer on the cell surface. The interaction of CD40L with CD40 effectively increases function of APCs, through the induction of costimulatory molecules as well as inflammatory cytokines and chemokines. Numerous reports have demonstrated that APCs that are activated as a result of the CD40L-CD40 interaction induce T cell activation [3, 4].

Together, these studies have supported the promise of CD40L-activated B cells for immunotherapeutic applications [5]. A widely available murine fibroblast cell line, engineered to stably express human CD40L (NIH3T3/tCD40L), has been shown to induce rapid expansion of normal human CD19+ B cells [6, 7]. These studies have provided proof-of-concept that CD40L-mediated stimulation can feasibly generate a large source of autologous APCs from relatively small amounts of peripheral blood. However, the presence of and response to mouse xenoantigens within the 3T3 cell system precludes its further clinical development. At the same time, despite the highly attractive immunostimulatory profile of CD40L, a purified soluble recombinant human formulation has not been readily available [8].

We sought to construct a soluble recombinant CD40L molecule that was sufficiently active to expand human B cells in vitro for use as APCs. A number of soluble recombinant forms of CD40L have been described with varying levels of bioactivity. These include formulations comprised of solely the TNF homology domain, of the TNF homology domain with the 75 amino acid stalk that joins the TNF domain to the membrane, and of each of the above joined to an isoleucine zipper trimerization motif [9, 10]. In addition, multimeric formulations of CD40L trimers have been made by linking CD40L to Acrp30 to produce a 2-trimer, or with the body of surfactant protein D to produce a 4-trimer protein [11, 12]. Some of these forms have short peptide linkers of 2–5 amino acids between the multimerizing motif and the CD40L. Studies have shown that the isoleucine zipper or the natural stalk domain do not increase CD40L bioactivity through trimerization, which is an intrinsic property of the TNF homology domain, but by stabilizing the structure by reducing unfolding of the protein [10]. In general, studies have shown that the greatest bioactivity is seen with stabilized trimers and multimerized trimers [9–12].

Since flexible peptide linkers of approximately 17 amino acids have been shown to optimally support folding of independent protein domains [13], we reasoned that inclusion of a long linker between the trimerization motif and the CD40L extracellular domain would best support CD40L bioactivity. We consequently used this approach to design a novel formulation of CD40L designated as CD40L-Tri. We demonstrate that CD40L-Tri readily self-multimerizes in solution and has potent immunostimulatory activity. These results lead us to speculate that the freedom of movement provided by the long peptide linker facilitates this process of self-multimerization and thereby enables effective cross-linking of CD40 on the cell surface.

## Materials and methods

### Patient samples

Heparinized peripheral blood was obtained from normal adult volunteers and patients enrolled on clinical research protocols at the Dana-Farber Harvard Cancer Center (DFHCC) approved by the DFHCC Human Subjects Protection Committee. Peripheral blood mononuclear cells (PBMC) were isolated by Ficoll/Hypaque density-gradient centrifugation and used fresh at the time of the experiment. Chronic lymphocytic leukemia cells were used fresh or cryopreserved with 10 % DMSO, and stored in vapor-phase liquid nitrogen until the time of analysis.

### Generation of CD40L-Tri

**Fig. 1:**
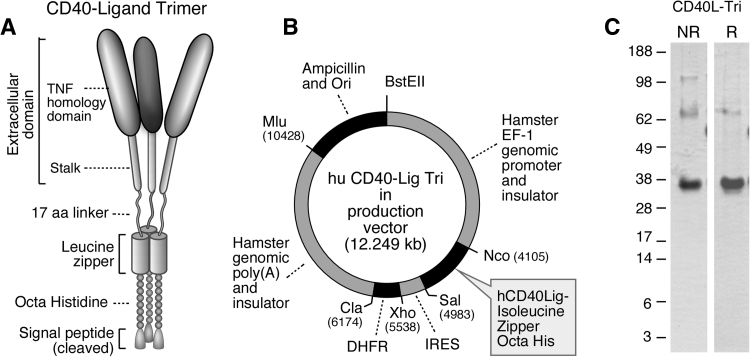
Generation of CD40L-Tri. Schematic of **a** the CD40L-Tri trimer, and of **b** the expression vector used to generate CD40L-Tri. **c** Non-reducing (NR) and reducing (R) SDS-PAGE of purified CD40L-Tri

The CD40L-Tri coding sequence is composed of an optimized IL-2 signal (MRRMQLLLLIALSLALVTNS) [14], an octahistidine, a trimeric leucine zipper GCN4pII heptad repeat derived from the wildtype dimeric GCN4 repeat found in *Saccharomyces cerevisiae* (GDRMKQIEDKIEEILSKIYHIENEIARIKKLIGER) [15], a flexible 17 amino acid linker (TSGGSGGTGGSGGTGGS) [13], and the extracellular domain of human CD40L (amino acids 51–261) [9]. The coding sequence was codon-optimized for CHO cell expression and synthesized by Genscript (Piscataway, NJ). The calculated molecular weight after signal cleavage is 30,093 daltons with a pI of 7.63. There is one predicted N-glycosylation site, which is expected to increase the molecular weight to the observed 35 kDa (Fig. 1c).

The CD40L-Tri insert was cloned into the pEFGF expression vector. The pEFGF vector is composed of 4.1 Kb 5′ of the hamster EF-1 gene including the EF-1 promoter, ending in a Kozak consensus start site that includes Nco I, EcoRV, and Sal I sites, followed by 0.5 Kb encephalomyocarditis (EMC) virus internal ribosome entry site (IRES) [16, 17], dihydrofolate reductase (DHFR) cDNA, the 4.3 kb hamster EF-1 polyadenylation and 3′ flanking sequence, ampicillin resistance, and origin of replication. This vector was based on the finding that non-promoter genomic elements in the hamster EF-1 locus support high-level protein expression independent of where the vector is integrated into the genome [18]. These non-promoter elements might include insulator elements, scaffold/matrix attachment regions, transcriptional enhancers, or terminators. The elements of the vector were generated by high-fidelity PCR with a unique restriction site and 15-bp overlap at the junction of each element, joined by In-Fusion [19], and confirmed by sequencing. The pEFGF vector was digested with Nco I and Sal I, and the CD40L-Tri coding sequence was amplified from the synthetic template with primers including 15-bp overlaps to the Nco I and Sal I sites of the vector and joined by In-Fusion. DHFR-deficient CHO cells (DG44) were electroporated with Mlu-linearized CD40L-Tri vector. After 2 days in non-selective media, cells were cultured in MEMα media without ribonucleosides and deoxyribonucleosides, 10 % dialyzed fetal bovine serum, 1 % glutamax, penicillin/streptomycin, and 15 mg/ml gentamicin. After 5 days, methotrexate was added to 25 nM and culture was continued until colonies grew out. Colonies were pooled and the concentration of methotrexate was increased in twofold steps to 1,000 nM, waiting for robust growth at each stage. Cells were subcloned at the 50 and 1,000 nM stages and selected for highest production level by ELISA. Cells were adapted to low-serum media, and culture supernatant was concentrated 12-fold by 50 kDa cut-off hollow fiber ultrafiltration (Spectrum Labs). Concentrated supernatant was passed through a column of His-Select HF Nickel Affinity Gel (Sigma), and the column was washed with 10 and 50 mM imidazole in 50 mM phosphate, 300 mM NaCl, pH 8.0, and eluted with 150 mM imidazole in the same buffer. The eluate was dialyzed twice against PBS, pH 7.0, and sterile filtered through 0.22 micron filter with a yield of 2.5 mg per liter. Of note, the 50 mM imidazole wash was necessary to remove a 55 kDa CHO cell protein, legumain. Endotoxin was determined by LAL gel assay to be less than 2 EU/mg protein.

### Other CD40L formulations

We tested 3 other formulations of human CD40L. Culture supernatants containing multimeric CD40L (‘Ultra-CD40L’, kindly provided by Richard Kornbluth, Multimeric, Inc, La Jolla, CA, USA) were produced by fusing the extracellular domain of CD40L with the body of surfactant protein D (a spontaneously multimerizing molecule) resulting in a 4-trimer soluble protein. We also tested a homotrimeric form of soluble human recombinant CD40L with a short linker, which we designated as ‘shrtCD40L’ (#2706-CL; R&D, Minneapolis, MN, USA). This form is only robustly active when cross-linked by monoclonal antibodies against the amino-terminal epitope tags (HexaHis or HA). As a positive control, we tested the natural membrane-bound form of human CD40L made by stable transfection of NIH3T3 cells, termed ‘NIH3T3/tCD40L’ [21].

### Expansion of human B cells with CD40L

CD19+ B cells were isolated from normal PBMC by immunomagnetic selection (CD19+ microbeads, Miltenyi, Auburn, CA, USA), and seeded at 2 × 10^6^ cells/well in a 24-well plate. Alternatively, the B cells were seeded at 0.3 × 10^6^ cells in 2 ml in a 25 cm^2^ cell culture flask. B cells were cultured in B cell media (Iscove’s modified Dulbecco medium (IMDM, Life Technologies)), supplemented with 10 % heat-inactivated human AB serum (Gemini Bioproducts, West Sacramento, CA, USA), 5 μg/ml insulin (Sigma Chemical, St Louis, MO, USA), 15 μg/ml gentamicin and IL-4 (2 ng/ml, R&D Systems, Minneapolis, MN, USA) supplemented with CD40L-Tri (at 1–10 μg/ml) [12, 20]. CD40L was replenished every 3–4 days. Alternatively, CD19+ B cells were activated and expanded using the established NIH3T3/tCD40L system, which acts as a CD40L-expressing irradiated feeder cell line, in media supplemented with IL-4 (R&D Systems) and cyclosporin A (Novartis, Basel, Switzerland) as described [6]. B cell expansion was measured by counting cells using trypan blue exclusion.

### Immunophenotyping and CFSE analysis

CD40L-activated B cells were evaluated using the following fluorophore-conjugated antibodies: CD80 (PE, Coulter; Fullerton, CA, USA); CD86 and CD83 (PE and PE-Cy5; BD Biosciences; San Jose, CA, USA); and CD19 (PC7, Beckman Coulter, Brea, CA, USA). B cells (0.5 × 10^6^) were washed twice with PBS and incubated with antibodies for 15 min at 4 °C. The stained cells were acquired on a FC500 instrument (Beckman Coulter, Brea, CA, USA). Analysis of immunophenotyping results was performed using FlowJo software (Tree Star, Inc. Ashland, OR). The relative expression of surface antigens was evaluated using the mean fluorescence intensity (MFI). To exclude the possibility that positive CD19+ B cell selection could by itself activate B cells, we performed CD40L-Tri expansion and immunophenotyping on negatively selected CD19+ B cells (B isolation Kit II, Miltenyi, Auburn, CA, USA). No discernable differences in results of CD40L activation were observed between positively and negatively selected B cells (Supplemental Figure 1). To examine cell division induced by CD40L-Tri, we labeled normal B cells with 5 μM CFSE (Invitrogen) for 10 min at 37 °C. 1 × 10^6^ of CFSE-labeled B cells were incubated with 1 μg/ml of CD40L-Tri. Activated B cells were harvested, stained with CD19-PC7 mAb, and analyzed for CFSE dilution by flow cytometry after 4, 7, and 10 days.

### T cell proliferation assay

Two days after activation by CD40L-Tri, Ultra CD40L, or short CD40L, B cells were irradiated (55 Gy) and used as stimulators (1–5 × 10^4^ cells/well) with immunomagnetically selected CD8+ or CD4+ allogeneic T cells (1 × 10^5^ cells/well; selected using CD8+ or CD4+ microbeads, Miltenyi Biotec, Auburn, CA, USA) in a final volume of 200 μl in 96-well round-bottom plates, at 37 °C in a 5 % CO_2_ humidified atmosphere. The cells were cultured in IMDM media supplemented with 10 % human serum, 2 mmol/L l-glutamine, 100 U/ml penicillin, and 100 μg/ml streptomycin. All co-culture conditions were performed in triplicate. After 5 days, 1 μCi [^3^H] thymidine (Amersham, PerkinElmer, Waltham, MA, USA) was added to each well. Cells were harvested onto glass fiber filters after 18–20 h, and [^3^H] thymidine incorporation was measured by scintillation counter.

### Generation and testing of M1 peptide-specific CD8+ T cells and CLL-reactive CD8+ T cells

CD8+ T cells specific for the influenza peptide M1 (GILGFVFTL, New England Peptide, Gardner, MA, USA) were generated from PBMC of HLA-A2+ normal volunteers. In brief, 2 × 10^6^ PBMCs were stimulated weekly with irradiated M1-pulsed APC (5 × 10^5^ T2 cells), or autologous B cells stimulated with CD40L-Tri (1 μg/ml) for a total of two stimulations, in the presence of 10 ng/ml IL-7 (R&D Systems, Minneapolis, MN, USA). In some experiments, peptide-pulsed autologous dendritic cells were used as APC and were generated as previously described [22]. All APCs were pulsed with peptide over 3 h and irradiated at 55 Gy. To detect the expanded M1-specific T cells, CD8+ T cells from the bulk co-cultures were immunomagnetically selected (CD8+ microbeads, Miltenyi, Auburn, CA, USA) and tested against a panel of target cells by ELISPOT assay. ELISPOT assays were performed as previously described [22]. In brief, target cells (50,000 cells/well) were co-incubated with 5,000 effectors on ELISPOT plates (Millipore, Billerica, MA, USA) in triplicate for 24 h. Interferon-γ secretion (IFNγ) was detected using capture and detection antibodies as directed (Mabtech AB, Mariemont, OH), and then subsequently imaged and quantified (ImmunoSpot Series Analyzer, Cellular Technology, Cleveland, OH). In other experiments, cryopreserved CLL cells were thawed, activated with 1 μg/ml CD40L-Tri, irradiated as described above, and co-cultured with autologous PBMCs. Ten days following stimulation, CD8+ T cells were immunomagnetically isolated, and tested for reactivity against CD40L-activated leukemia cells by ELISPOT assay.

### Biochemical characterization

To identify the active form of the CD40L-Tri, 400 μg was buffer-exchanged into HEPES-saline (150 mM NaCl, 20 mM HEPES; pH 7.48), and separated by size-exclusion chromatography on a Superdex 200 HR10/300 analytical column (~25 ml bed volume, HEPES-saline running buffer) inline in an ÄKTAexplorer 10 FPLC system (GE Healthcare Life Sciences, Piscataway, NJ). Fractions of 0.5 ml were collected, and analyzed on reducing SDS-PAGE and native gels by silver staining (Thermo Scientific, Rockford, IL). After sterilization by filtration (Spin-X), these fractions were tested in tissue culture for upregulation of B cell costimulatory markers. Protein content, initially evaluated by absorbance at 280 nm, was determined using the microBCA assay (Thermo Scientific) as the low aromatic residue content of CD40L-Tri results in a low molar extinction coefficient (for monomer, ε = 14,440 M^−1 ^cm^−1^).

### Statistical analysis

A one-sided paired Student’s *t*-test was used to test for an increase from baseline for each treatment groups for the expression of costimulatory molecules and considered significant if* p* < 0.05. For the T cell proliferation experiments, a 2-way ANOVA model was constructed and included an interaction term for dilution and treatment effect. Longitudinal models were constructed for repeated measures of the ratio of cell number to baseline over time and included an interaction term for treatment group and time. A compound symmetry covariance structure was used for these models. All *p* values provided for longitudinal and ANOVA modeling were Bonferroni adjusted and considered significant if <0.05.

## Results

### Generation of CD40L-Tri

CD40L-Tri was designed as a self-assembling homotrimer and consists of a signal sequence, octahistidine motif for purification, isoleucine zipper for trimerization, long flexible linker of 17 amino acids, and the extracellular domain of CD40L (Fig. 1a). CD40L-Tri was produced in CHO cells (Fig. 1b) and purified material consisted primarily of a 35 kDa protein on reducing SDS-PAGE. Non-reducing gels showed higher molecular weight bands consistent with dimeric and trimeric forms (Fig. 1c).

### Stimulation of B cells by CD40L-Tri upregulates expression of costimulatory molecules

**Fig. 2:**
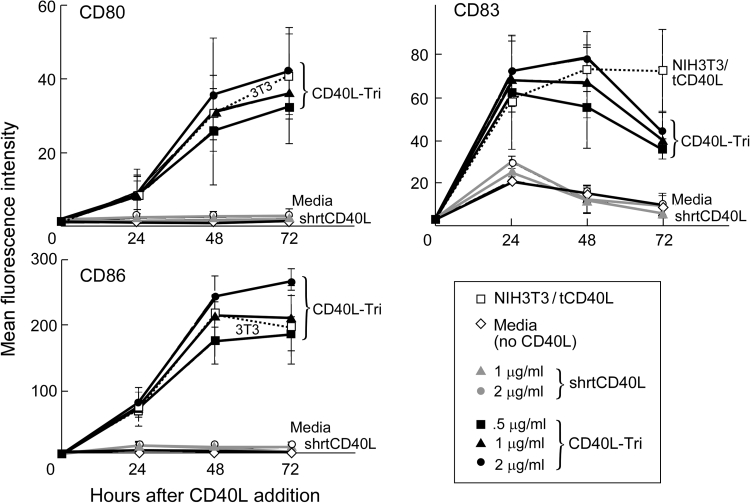
CD40L-Tri upregulates expression of costimulatory molecules on normal B cells. CD19+ B cells isolated from normal volunteers (*n* = 3) were cultured with various formulations of CD40L, including CD40L expressed on transfected cells (irradiated NIH3T3/tCD40L), a recombinant homotrimer with a short linker (shrtCD40L, *gray symbols*), and the test recombinant protein with a long flexible linker (CD40L-Tri, *black symbols*). Cells were evaluated by flow cytometry for expression of CD80, CD83, and CD86 at the indicated times. The mean MFI and *error bars* (±SD = standard deviation) for each treatment group are shown at each time point

Many of the immunostimulatory consequences of CD40L activation of B cells are mediated through its ability to upregulate expression of costimulatory molecules such as CD80, CD83, and CD86. To examine the potency of CD40L-Tri, we measured its ability to enhance expression of these molecules compared with known formulations of CD40L, namely the transfected murine fibroblast line (NIH3T3/tCD40L) and commercially available epitope-tagged homotrimeric forms that are formulated with a short linker (designated ‘shrtCD40L’) and that are inactive unless cross-linked by tag-specific antibodies. Culture of normal CD19+ B cells with CD40L-Tri (at 2 μg/ml; *n* = 3) consistently resulted in expression of CD80, CD83, and CD86 at 48 h with mean increases of 29-, 24-, and 165-fold, respectively, (*p* < 0.05) which was comparable to NIH3T3/tCD40L (25-, 22-, and 146-fold, respectively; *p* < 0.05) (Fig. 2). Seventy-two hours following exposure to CD40L-Tri (2 μg/ml), the expression of CD80 and CD86 persisted (mean of 34- and 180-fold) while expression of CD83 decreased to 13-fold increase from baseline. No increase in expression of costimulatory molecules was observed following exposure to shrtCD40L, as expected without cross-linking.

### CD40L-Tri-activated B cells induce proliferation of allogeneic CD4+ and CD8+ T cells

**Fig. 3:**
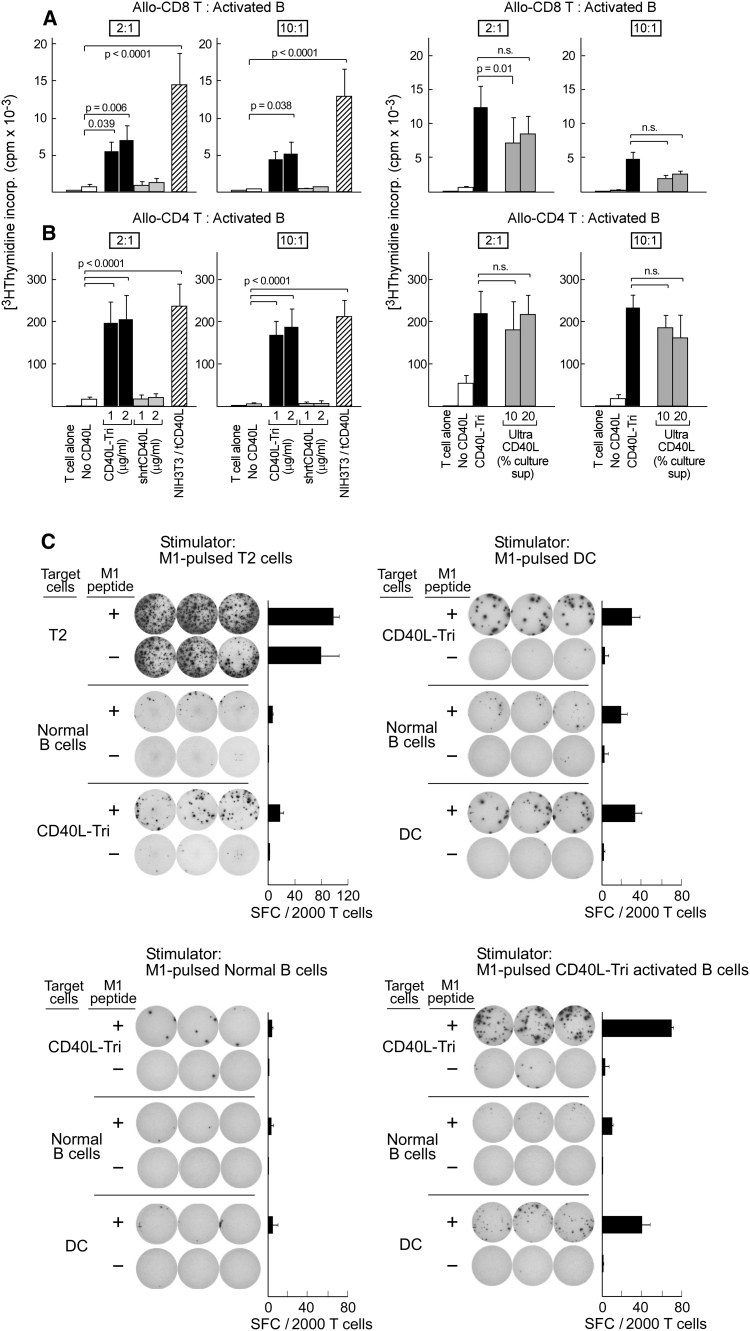
CD40L-Tri generates activated B cells that strongly stimulate expansion of allogeneic CD8+ and CD4+ T cells. CD19+ B cells were cultured without CD40L (‘no CD40L’) or with various formulations of CD40L, including a recombinant homotrimer with a long flexible linker (‘CD40L-Tri’), a recombinant homotrimer with a short linker (‘shrtCD40L’), a recombinant 4-trimer form (‘Ultra CD40L’), or irradiated NIH3T3/tCD40L. Activated B cells were harvested after 2 days, irradiated, and co-cultured with allogeneic CD8+ T cells (**a**) or CD4+ T cells (**b**) at the indicated ratios. T cell proliferation was measured after 5 days. The figure displays the mean and *error bars* (+SD) for each treatment group from 3 adult volunteers assayed in triplicate. **c** Representative results (of experiments from 3 donors) of generation of M1 peptide-specific CD8+ T cells from a HLA-A2+ donor, by stimulation of T cells with M1 peptide (10 μg/ml) loaded on T2 cells, CD40L-Tri-activated B cells, normal B cells, or DCs. After 1 week, expanded CD8+ T cells (5,000) were co-cultured with the indicated target cells with or without M1 peptide for 24 h on IFNγ ELISPOT plates in triplicate

Since CD40L-Tri clearly induced expression of costimulatory molecules on B cells, we next investigated the capacity of CD40L-Tri-activated B cells to stimulate proliferation of allogeneic T cells. B cells were activated with CD40L-Tri or other CD40L formulations (shrt-CD40L, NIH3T3/tCD40L, Ultra-CD40L), irradiated, and co-cultured at a ratio of 2:1 or 10:1 with allogeneic CD4+ or CD8+ T cells. T cell proliferation was measured by [^3^H] thymidine incorporation at 5 days following the initiation of co-culture. CD40L-Tri-activated B cells (used at 1–2 μg/ml) consistently stimulated proliferation of CD8+ T cells from three normal volunteers (Fig. 3a, right panel). At a 2:1 ratio, CD8+ T cells, when exposed to allogeneic CD40L-Tri-activated B cells, proliferated 8–11-fold more than when exposed to non-activated B cells (*p* < 0.05), and 6–8-fold more than when exposed to B cells treated with shrt-CD40L (1 μg/ml) (*p* < 0.05), which is known to be non-bioactive in the absence of a cross-linking antibody. These results compared favorably to the extent of proliferation elicited by NIH3T3/tCD40L-activated B cells (22-fold more than non-activated B cells; *p* < 0.05). Similarly, as shown in Fig. 3b (right panel), we observed high proliferation of allogeneic CD4+ cells when co-cultured with irradiated CD40L-Tri-activated B cells at 2:1 ratio (12–13-fold, compared with either shrt-CD40L or non-activated B cells) that was comparable to stimulation by NIH3T3/tCD40L-activated B cells (14-fold). We also established that the stimulatory capacity of CD40L-Tri-activated B cells was as strong as B cells activated by a multimerized CD40L (Ultra-CD40L) [11] (Fig. 3a, b left panel). Together, these results demonstrate that CD40L-Tri has potent immunostimulatory activity.

### CD40L-Tri-activated B cells are effective stimulators and targets of antigen-specific T cells

We next confirmed that CD40L-Tri could stimulate expansion of CD8+ T cells in an antigen-specific manner, using the well-characterized HLA-A2+ restricted influenza M1 peptide (GILGFVFTL) as immunogen. HLA-A2+ PBMC were stimulated with M1-pulsed autologous CD40L-Tri B cells, non-activated B cells, dendritic cells, or the HLA-A2-expressing T2 cell line. Following two weekly stimulations with the M1 peptide, CD8+ T cells were isolated from the bulk cultures and specificity for M1 peptide was tested by IFN-γ ELISPOT. CD40L-Tri-activated B cells displayed comparable immunostimulatory capacity as dendritic cells and were far superior to non-activated B cells (Fig. 3c). In addition, CD40L-Tri-activated B cells stimulated T cell expansion with much better specificity than T2 cells. Furthermore, peptide-pulsed CD40L-Tri-activated B cells functioned well as targets of antigen-specific T cells (Fig. 3c).

**Fig. 4:**
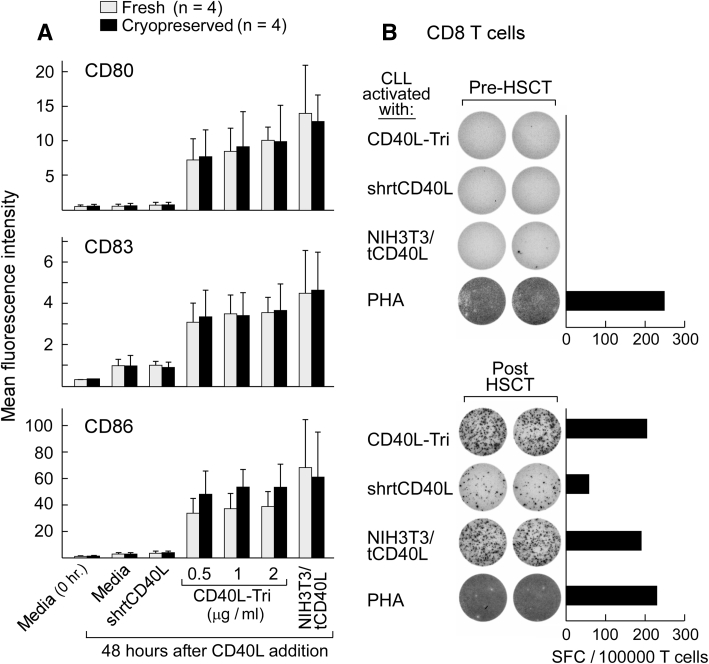
CD40L-Tri-activated CLL cells effectively elicit patient CD8+ T cell responses. **a** Tumor cells freshly isolated from CLL patients (*n* = 4) were directly cultured with CD40L-Tri, irradiated CD40L-expressing NIH3T3 cells (NIH3T3/tCD40L), or recombinant CD40L (shrtCD40L) (*open bars*) or initially cryopreserved, thawed, and then incubated with the various CD40L formulations (*black bars*). Forty-eight hours later, the expression of CD80, CD83, and CD86 was evaluated by flow cytometry. The mean MFI and *error bars* (±SD) for each treatment group are depicted. **b** PBMCs from a patient with chronic lymphocytic leukemia (CLL) before hematopoietic allogeneic stem cell transplantation (‘pre-HSCT’) (top) and following transplant (‘post-HSCT’) (*bottom*) were stimulated with CD40L-Tri-activated autologous CLL cells, and immunomagnetically selected CD8+ T cells were restimulated on ELISPOT assay with autologous CLL cells activated by the indicated CD40L formulation. The mean number of IFNγ-specific spots is displayed for each group

We further tested the capacity of CD40L-Tri to increase the immunostimulatory activity of malignant B cells. Chronic lymphocytic leukemia (CLL), a disease of clonal CD19+ CD5+ CD23+ B cells, is sensitive to immune modulation. The development of methods to stimulate and monitor immunity against these leukemia cells, however, has been impaired by the poor antigen-presenting ability of CLL cells due to their low expression of costimulatory molecules. These defects can be reversed through stimulation with CD40L, for example, through use of the widely used NIH3T3/tCD40L system [23]. We observed that like normal CD19+ B cells, CLL cells (either fresh or cryopreserved) gain enhanced expression of costimulatory molecules following CD40L-Tri exposure (Fig. 4a, Supplemental Figure 2). We therefore tested a series of PBMC samples collected from CLL patients before and after allogeneic hematopoietic stem cell transplantation (HSCT) for the development of anti-CLL immunity after HSCT. These patients demonstrated molecular remission following HSCT. As shown in the example in Fig. 4b, patient PBMC that were collected 6 months following HSCT were co-cultured with CD40L-Tri-activated autologous CLL cells, and then purified CD8 T cells were restimulated in an IFNγ-ELISPOT assay with autologous CLL cells activated by CD40L-Tri, NIH3T3/tCD40L, or shrtCD40L, or by PHA. CD40L activation of CLL cells by the CD40L-transfected cell line or by CD40L-Tri elicited equivalently high reactivity, while the shrtCD40L-activated tumor targets elicited detectable but weaker reactivity. Patient PBMC collected before HSCT had minimal reactivity.

### CD40L-Tri elicits expansion of B cells

**Fig. 5:**
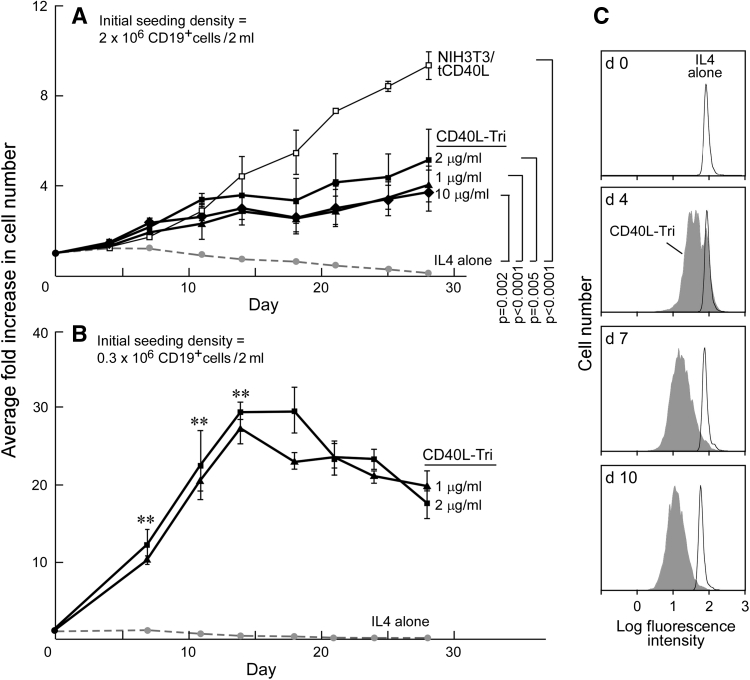
CD40L-Tri can effectively expand normal B cells. Average fold increase in B cell number, when CD19+ B cells were seeded at **a** high density (2 × 10^6^ CD19+ B cells/2 ml) and **b** low density (0.3 × 10^6^ CD19+/2 ml). B cells were incubated with the indicated CD40L formulation as described in ‘Materials and methods’, counted on the indicated days and the ratio of cell number to baseline was calculated, with *each line* representing the mean ± SD over time, obtained from testing of cells from 3 adult volunteers. In (**a**), the significance is only shown for day 28 based on modeling for each group versus IL-4 alone, and the trend over all time points was significantly different for each group versus IL-4, *p* < 0.001. **p < 0.001 for each CD40L-Tri group compared with IL-4 alone. **c** CFSE-labeled normal B cells were stimulated with CD40L-Tri and cell division measured by CFSE dilution (*shaded*) by flow cytometry, compared with non-activated cells (‘IL4 alone’, *non-shaded*)

The ability of human CD40L to persistently expand normal B cells has been described [7, 11] and establishes the potential of using this molecule to generate a renewable source of antigen-presenting cells from primary human cells. We initially compared the ability of CD40L-Tri and NIH3T3/tCD40L cells to induce and sustain proliferation of B cells in high-density cultures (2 million cells/2 ml initial seeding density). As expected, we observed a lack of expansion of CD19+ B cells in the presence of IL-4-containing media without CD40L, and a ninefold mean expansion with NIH3T3/tCD40L cells at day 28 (Fig. 5a). CD40L-Tri (1, 2, and 10 μg/ml, replenished every 3–4 days) stimulated B cells expanded by 4–5-fold at day 28 compared with IL-4 (0.10-fold) (*p* < 0.05); however, this was less than the expansion with NIH3T3/tCD40L cells. These results were not altered by addition of conditioned media from NIH3T3 cells together with CD40L-Tri into the culture media, excluding the idea that the NIH3T3 cells provided soluble factors that contributed to B cell proliferation (data not shown).

On the other hand, we observed much more expansion when B cell cultures were initiated at low density (0.3 × 10^6^ cells/2 ml seeding density) (Fig. 5b). At this approximately tenfold lower seeding density, CD40L-Tri (2.0 μg/ml) stimulation resulted in a 20- to 30-fold mean expansion at 14 days. At day 28, the rate of expansion decreased to a mean of 18-fold. Notably, analysis of B cell division by CFSE dilution demonstrated proliferation within the entire population of B cells (Fig. 5c).

The favorable growth kinetics in low initial B cell seeding density between the soluble formulations and the CD40L-expressing murine fibroblast line likely reflects the difference in CD40L bioavailability when provided as soluble recombinant form or as a continuously renewing membrane source from a cell line.

### Bioactivity of CD40L-Tri is related to self-multimerization

**Fig. 6:**
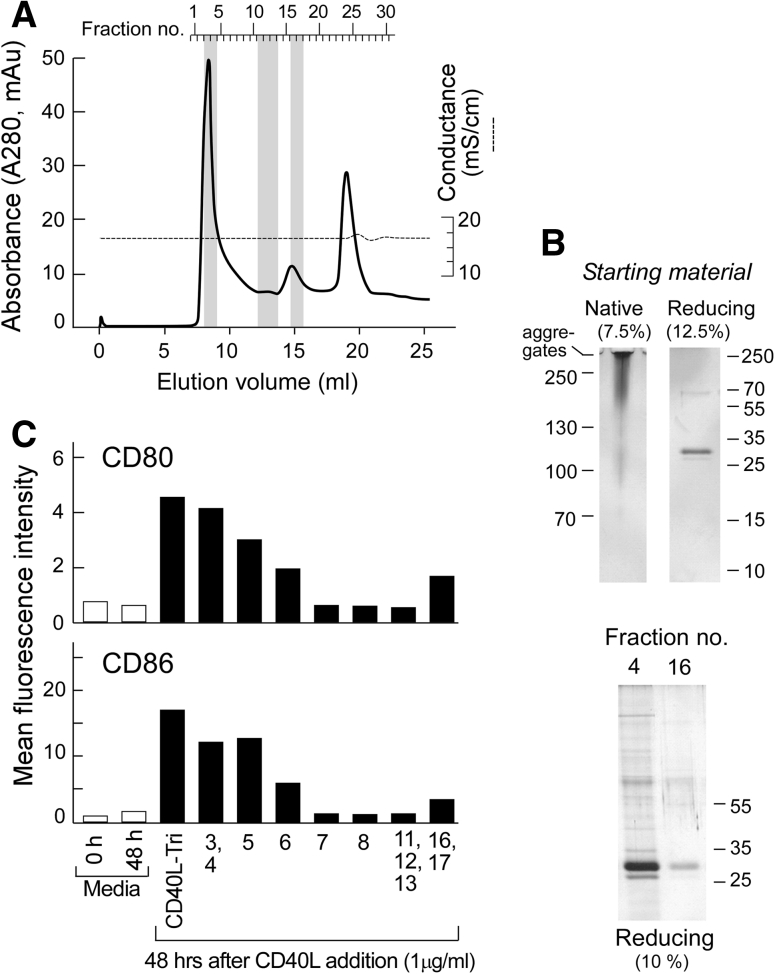
Bio-active CD40L-Tri is predominantly a multimer. **a** Superdex 200 HR10/300 separation of CD40L-Tri. OD_280_ plot of fractions from size-exclusion gel chromatography of CD40L-Tri (*solid line*). Elution volume is presented on the lower abscissa and collected fraction number on the upper abscissa. Protein was collected at 0.5 ml elution volumes, and the end of the run is indicated by the disturbance in the conductance (*dashed line*) at 20 ml elution volume. The main peak (fractions #3–6) and second peak (fractions #15–17) represent separated CD40L-Tri protein. The peak at fractions #24–25 is non-proteinaceous UV-absorbing material. **b** Upper panel, left—native gel electrophoresis in glycine buffer (pH 8.8; 7.5 % acrylamide gel) of unseparated CD40L-Tri. Note that the protein was applied in glycerol-free, sucrose-free loading buffer as both agents led to complete aggregation and no entry into the gel. Upper panel, right—reducing SDS-PAGE analysis (12.5 % acrylamide gel) of unseparated CD40L-Tri indicates that the variably sized entities identified on the native gel are all comprised of a monomer of ~35 kDa. Lower panel—reducing SDS-PAGE analysis (10 % acrylamide gel) of fractions 4 and #16 indicates that they are both comprised of the same base ~35 kDa monomer. **c** Normal CD19+ B cells were incubated with individual or pooled chromatographic protein fractions at a concentration of 1 μg/ml. Forty-eight hours later, the expression of CD80 and CD86 on the cell surface was evaluated by flow cytometry. The stimulatory capacity of fraction pools 3, 4 and 16, 17 as well as the unseparated material (CD40L-Tri) and the media control were analyzed in duplicate. The mean MFI is shown for each treatment group

Since we observed that CD40L-Tri bioactivity was similar to that of a multimerized CD40L, we queried whether CD40L-Tri was aggregating in solution and which molecular size fraction exhibited the greatest bioactivity. Size-exclusion gel chromatography (SEC) revealed that CD40L-Tri eluted primarily in a single peak (fractions #3 and #4, eluting at 8–9 ml coinciding with the void volume and slightly after) consistent with a molecular weight range of ~400 kDa and higher. A second smaller peak eluted in fractions #15–17 (eluting at 14–15.5 ml) and corresponded to a molecular weight of ~65 kDa (Fig. 6a).

We consistently noted across experiments using several columns that CD40L-Tri exhibits secondary interactions with the Superdex matrix retarding its elution leading to an underestimation of the nominal molecular weight. As a consequence, we ran a native gel in a glycine-based system (pH 8.8; to ensure that CD40L-Tri was negatively charged). We observed that the unseparated starting material appeared as a smear initiating at ~90–100 kDa that gradually increased to large aggregates that did not enter the gel (Fig. 6b, left). On the other hand, reducing gel analysis of the CD40L-Tri starting material clearly indicated that the dominant component is the CD40L monomer (Fig. 6b, right). Furthermore, similar analysis of fractions 4 and 16 indicated they consist of identical components of ~35 kDa in size, consistent with the CD40L monomer. Our results thus support the conclusion that the majority of the material in CD40L-Tri is contained in the first eluting peak and consists of higher-order oligomers of the material in the second eluting peak (which appears to consist of the CD40L-Tri base trimer).

Comparison of the bioactivity of the unseparated and fractionated material revealed that pooled fractions 3 and 4 showed a comparable strong stimulatory capacity as the starting material and that the following chromatographic fractions exhibited a gradual decline in biological activity. Pooled fractions collected from the second eluting peak (fraction 16 and 17) moderately induce cell surface expression of CD80 and CD86 in CD19+ B cells, supporting the notion that this eluting peak contains single CD40L-Tri timer. However, the biological activity of the unseparated CD40L-Tri material is predominantly maintained within the first eluting peak, suggesting that the potent immune-stimulatory effects of CD40L-Tri on B cells are constituted by self-multimerization.

## Discussion

It has long been appreciated that CD40L is physiologically formulated on the cell surface in vivo as a homotrimer. Growing evidence, however, suggests that homotrimerization alone is necessary but not sufficient for maximal activity [11]. Rather, higher-order clustering of CD40 appears to be required for optimal CD40 signaling. These concepts have been supported by studies demonstrating that multimeric forms of CD40L homotrimers were more active than homotrimers alone [9–12]. More recently, the mechanism underlying the activity of agonistic CD40 antibodies has been elucidated and shown to rely on the clustering of mAb-FcR units [24]. In the current manuscript, we describe a novel formulation of a highly active single trimer recombinant formulation, CD40L-Tri, that is constructed using a long flexible linker. We have demonstrated that CD40L-Tri efficiently upregulated costimulatory molecules on B cells, dramatically induced B cell expansion, and that these activated B cells could stimulate T cell responses.

We found that the high bioactivity of our CD40L-Tri appears to depend on multimerization of trimers. Generally, TNFR family members are monomers that interact with a TNF family member by a single TNFR binding in each of the 3 interfacial grooves formed by trimerization of TNF, thus generating a 3:3 complex [25]. Recently, crystallography studies have demonstrated that the co-crystal structure of CD40:CD40L unexpectedly showed a 2:3 stoichiometry with a CD40 occupying only 2 of the 3 available CD40L grooves [26]. We therefore speculate that the addition of the long 17 aa linker in the CD40L-Tri molecule provides an extra degree of freedom that enables effective CD40 signaling on cell surfaces.

CD40L-Tri is straightforward to manufacture, and could be readily applied for investigative studies in immunotherapy and B cell biology. We demonstrate that CD40L-Tri-activated B cells can generate autologous APCs with equivalent potency as dendritic cells, but have the advantage that little starting material is needed. Compared with the conventionally available NIH3T3/tCD40L cells, using recombinant CD40L-Tri is far less labor intensive, easier to control, and eliminates the xenoantigen exposure that can confound studies of T cell reactivity. We also show that CD40L-Tri-activated B cells function well as targets of T cell responses. On the other hand, there is a risk of expanding Tregs using CD40L [27]; however, we did not see evidence of this in our M1-peptide experiments that started with PBMC.

A high priority for the field of cancer immunotherapy is the increased availability of potent immunostimulatory agents for enabling the clinical development of immune treatments [8]. CD40 and CD40L (CD154) have been ranked among the 10 most important immunomodulatory agents. Because of the ease of manufacture, CD40L-Tri can be readily applied to the clinical setting, with potential applications for cancer vaccines, adoptive immunotherapy, and for the monitoring of immune responses.

## Electronic supplementary material

Below is the link to the electronic supplementary material.

Supplemental Figure 1: Positive B cell selection does not result in pre-activation of B cells. B cells were either separated via CD19-specific immunomagnetic beads (positive selection; black lines) or isolated as untouched B cells (negative selection; blue lines) from PBMCs of healthy volunteers (*n* = 3). Following a 24- or 48-h culture with CD40L-Tri, cell surface expression of CD80 and CD86 was evaluated by flow cytometry. For each treatment group, the mean MFI (±SD) is depicted

Supplemental Figure 2: CD40L-Tri enhances the surface expression of costimulatory molecules on fresh and cryopreserved CLL cells. (**A**) Freshly isolated (*n* = 4) or (**B**) initially cryopreserved CLL cells (*n* = 4) were cultured with various CD40L formulations (CD40L-Tri: *filled black symbols*; irradiated NIH3T3/tCD40L cells: *open symbols*; shrtCD40L: *gray symbols*) as described in Figure 4. The cell surface expression of CD80, CD83, and CD86 was evaluated at 0, 24, 48, and 72 h following treatment by flow cytometry. The mean MFI (±SD) is shown for each treatment group
