# Supplementary figures and images for: CD40L-Tri, a novel formulation of recombinant human CD40L that effectively activates B cells
Source: Cancer Immunol Immunother. 2012 Aug 25;62(2):347–57. doi: 10.1007/s00262-012-1331-4 (PMC3569584; doi:10.1007/s00262-012-1331-4)

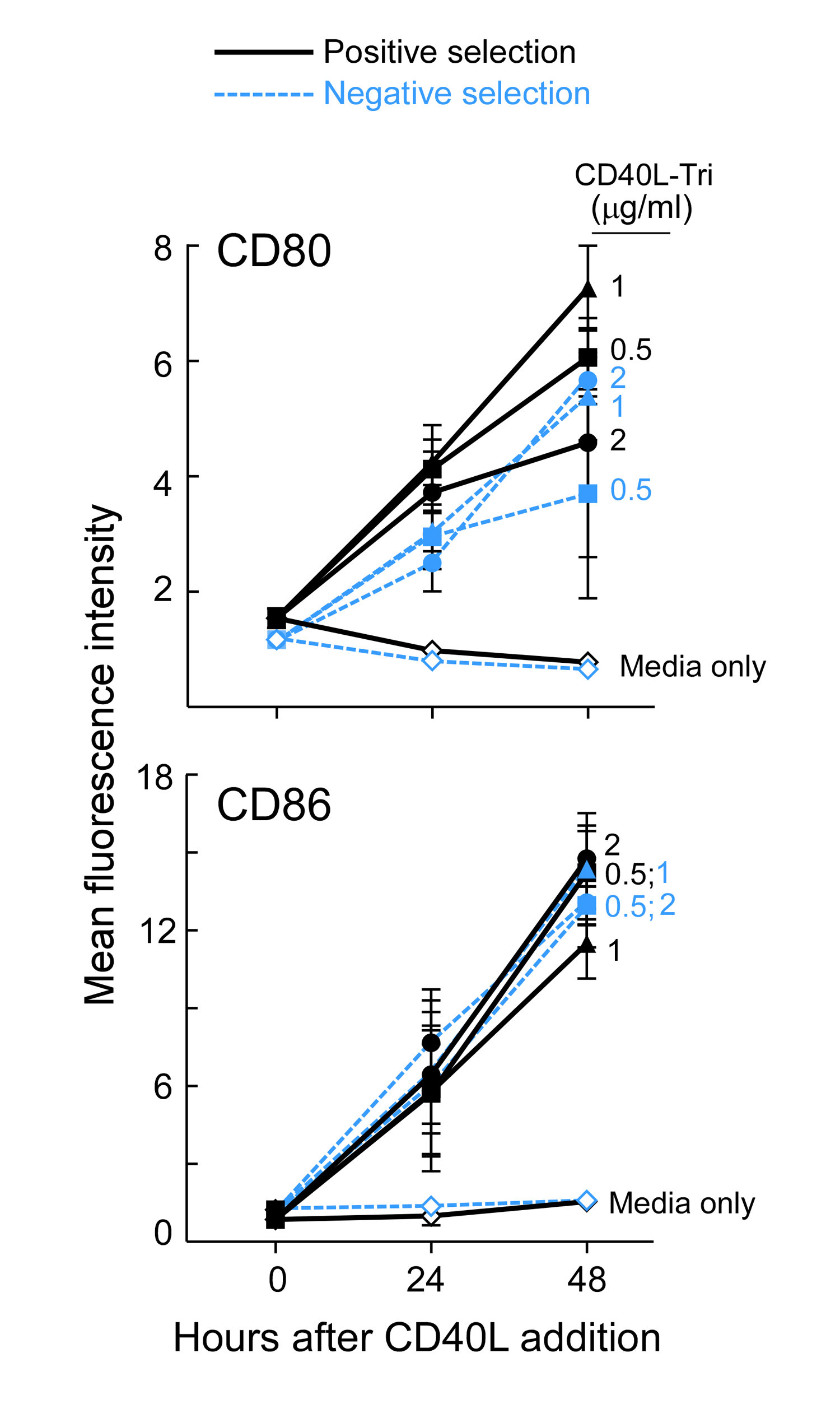

Supplement: Supplementary file 1 — Supplemental Figure 1: Positive B cell selection does not result in pre-activation of B cells. B cells were either separated via CD19-specific immunomagnetic beads (positive selection; black lines) or isolated as untouched B cells (negative selection; blue lines) from PBMCs of healthy volunteers (n = 3). Following a 24- or 48-h culture with CD40L-Tri, cell surface expression of CD80 and CD86 was evaluated by flow cytometry. For each treatment group, the mean MFI (±SD) is depicted [file 262_2012_1331_MOESM1_ESM.tif]

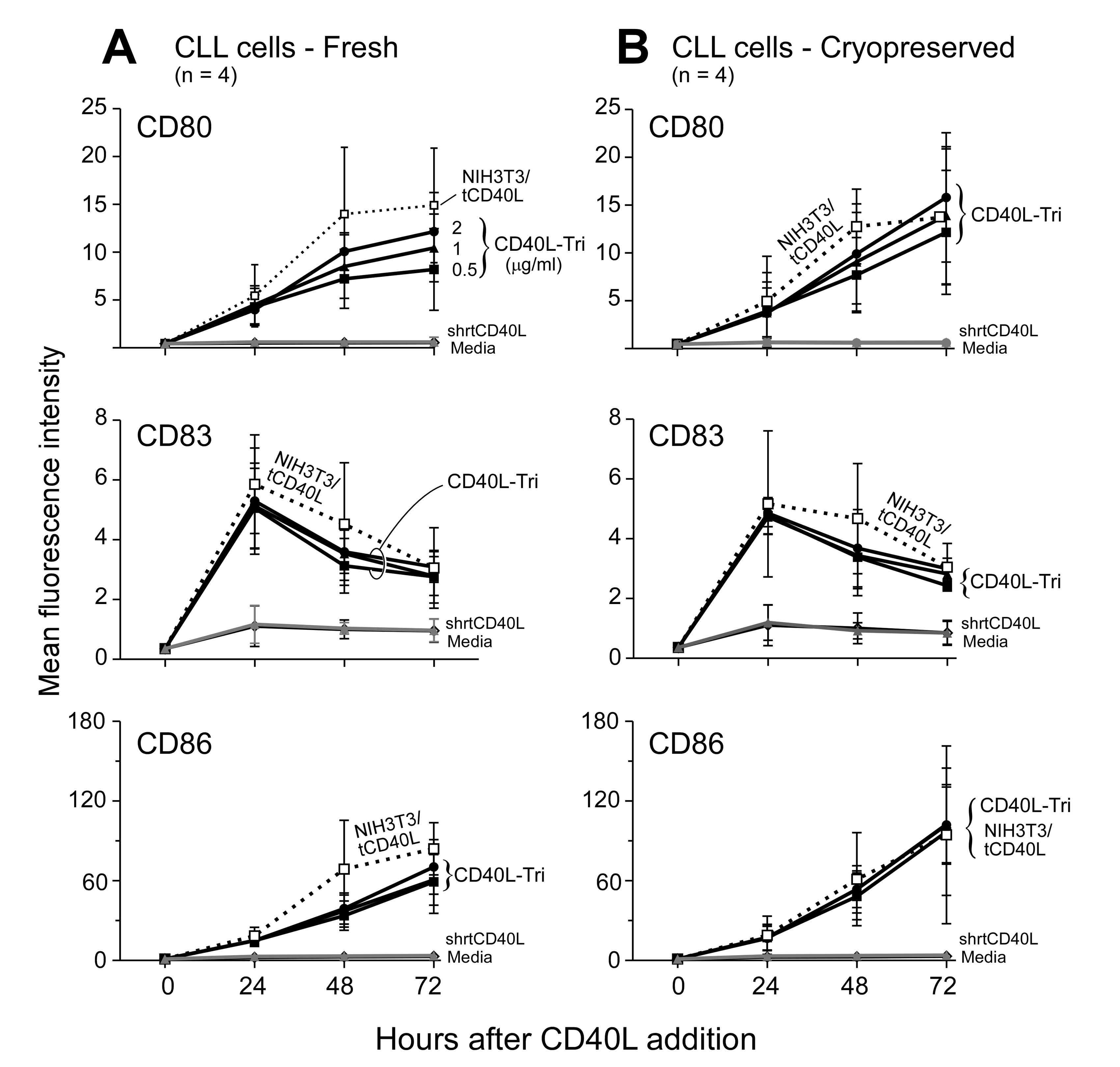

Supplement: Supplementary file 2 — Supplemental Figure 2: CD40L-Tri enhances the surface expression of costimulatory molecules on fresh and cryopreserved CLL cells. (A) Freshly isolated (n = 4) or (B) initially cryopreserved CLL cells (n = 4) were cultured with various CD40L formulations (CD40L-Tri: filled black symbols; irradiated NIH3T3/tCD40L cells: open symbols; shrtCD40L: gray symbols) as described in Figure 4. The cell surface expression of CD80, CD83, and CD86 was evaluated at 0, 24, 48, and 72 h following treatment by flow cytometry. The mean MFI (±SD) is shown for each treatment group [file 262_2012_1331_MOESM2_ESM.tif]
